# 3D Gravity and magnetic inversion modelling for geothermal assessment and temperature modelling in the central eastern desert and Red Sea, Egypt

**DOI:** 10.1038/s41598-024-65131-z

**Published:** 2024-07-03

**Authors:** Gaber M. Gaber, Salah Saleh, Adel Kotb

**Affiliations:** 1 Geology Department, Faculty of Science, Helwan University, Ain Helwan, Cairo, Egypt; 2 National Research Institute of Astronomy and Geophysics (NRIAG), Helwan, Cairo, Egypt

**Keywords:** Central Eastern Desert, Red Sea, Bouguer Gravity, Magnetic, Inversion modelling, Geothermal potential, Geodynamics, Geophysics

## Abstract

The Central Eastern Desert and Red Sea region have emerged as a significant area of interest for geothermal energy exploration, owing to their unique geological characteristics and active tectonic activity. This research aims to enhance our understanding of the region's geothermal potential through a comprehensive analysis of gravity and magnetic data. By utilizing a 3D gravity inversion model, a detailed examination of subsurface structures and density variations was conducted. Similarly, a 3D magnetic inversion model was employed to investigate subsurface magnetic properties. Integration result from the Pygimli library ensured robustness and accuracy in the inversion results. Furthermore, a temperature model was developed using the WINTERC-G model and inversion techniques, shedding light on the thermal structure and potential anomalies in the study area. The analysis of the Bouguer gravity map, 3D gravity inversion model, and magnetic data inversion yielded significant findings. The Red Sea exhibited higher gravity values compared to the onshore Eastern Desert, attributed to the presence of a thinner and denser oceanic crust as opposed to the less dense continental crust in the Eastern Desert. The 3D gravity inversion model revealed distinct variations in density, particularly high-density zones near the surface of the Red Sea, indicating underlying geological structures and processes. Conversely, density gradually decreased with depth along the onshore line, potentially influenced by a higher concentration of crustal fractures. The magnetic data inversion technique provided additional insights, highlighting areas with demagnetized materials, indicative of elevated temperatures. These findings were consistent with the correlation between high-density areas and low magnetic susceptibility values, reinforcing the proposition of increased heat transfer from the Red Sea. Comparative analysis of temperature profiles further confirmed the presence of elevated temperatures in promising zones, emphasizing the geothermal potential associated with heat transfer from the Red Sea.

This research contributes to the understanding of the geothermal resources in the Central Eastern Desert and Red Sea region. The results from gravity and magnetic data inversions, combined with temperature profiles, provide valuable information for future geothermal exploration and utilization efforts. The findings underscore the importance of geothermal energy in achieving sustainability and contribute to the global discourse on renewable energy sources.

## Introduction

The Central Eastern Desert and Red Sea region are renowned for their remarkable geological features and tectonic activity, which have attracted significant attention to their potential for geothermal energy resources. This area serves as a captivating subject for research and exploration, holding immense promise for sustainable energy development. To effectively assess and harness these valuable resources, it is crucial to comprehend the region's geothermal history, previous studies conducted in the area, and the existing temperature models.

The presence of the Red Sea Rift, a major tectonic feature resulting from the separation of the Arabian and African plates, characterizes the Central Eastern Desert and Red Sea region. This ongoing tectonic activity has contributed to the formation of volcanic features and the existence of geothermal phenomena. This complex geological setting within this region provides favourable conditions for the occurrence of geothermal resources, making it an ideal location for further exploration and utilization.

Over the years, numerous studies have been conducted in the Central Eastern Desert and Red Sea region to evaluate its geothermal potential and gain insights into the distribution of subsurface temperatures. These studies have played a pivotal role in enhancing our understanding of geothermal locations and areas of interest and have provided valuable information for guiding future geothermal exploration and utilization efforts^1–10^.

One particularly noteworthy aspect of geothermal research in this area is the development of temperature models that map the thermal characteristics of the subsurface. These models are constructed by assimilating data from various sources, including geological surveys, geophysical measurements, and borehole data. By analyzing the temperature distribution at different depths, these models offer critical insights into the presence of geothermal resources and potential reservoirs within the region.

The present research builds upon the foundation laid by previous studies, The first studies conducted on the geothermal potential in Egypt were those of Morgan et al.^1^ and Boulos^2^. They observed hot springs with temperatures as high as 76 °C in various locations along the western and eastern Gulf coastlines, where active structural systems in the Gulf of Suez area were related to block faulting. The heat of these springs is likely generated by intense heat flow (HF) and deep circulation controlled by faults related to the Gulf of Suez rift and Red Sea opening. They analyzed the temperature from the surface thermal water and many deep oil wells and provided detailed thermal gradient (TG) and HF mapping of northern Egypt. The potential of geothermal resources on an industrial scale was further researched by Boulos^3^. He proposed the latest plan for using ocean thermal energy conversion to provide 150–200 kW of electricity needed for the growth of the tourism industry, using a single-stage binary plant using ammonia fluid.

Chandrasekharam et al.^4^ have researched geothermal systems around the Eastern Desert and the Gulf of Suez, which are characterized by rock types with granitic composition with high heat-generating potential. The RHP (Radioactive Heat Production) (expressed in milliwatts per cubic meter) of these granites was determined using the constant of heat generation (per gram of uranium [U], thorium [Th], and potassium [K] released heat per unit time) and amount of radioactive materials (U, Th, and K). The average HF value through the granite was 220 mW/m2, and the average value of heat production was 18 mW/m3. The newer granite in the Eastern Desert includes secondary uranium deposits and uranium-rich mineral phases, which may be the source for these high values.

Previous studies have been conducted in other areas of Egypt. Abdel Zaher et al. ^5^ conducted a geothermal exploration using airborne gravity and magnetic data at Siwa Oasis in the Western Desert of Egypt. Mohamed et al.^6^ developed a geothermal gradient map of the Northern Western Desert of Egypt using aerogravity and BHT (Bottom Hole Temperature) data and an artificial neural network. Abdel Zaher et al.^7^ explored and assessed geothermal resources in the Hammam Faraun hot spring in the Sinai Peninsula, while Abdel Zaher et al.^8^ conducted a preliminary regional geothermal assessment of the Gulf of Suez. Abdel Zaher et al.^9^ studied the geophysical structures of several geothermal fields with conceptual and numerical models of the hottest spring in Egypt. Mohamed et al.^10^ developed conceptual and numerical models of the Hammam Faraun hot spring in the Sinai Peninsula.

This study utilized various geophysical techniques, including aeromagnetic and gravity data, and inversion modelling to explore and assess geothermal resources in the study area. The study aimed to provide a better understanding of the thermal structure of the region and identify potential geothermal resources for renewable energy production.

This research aligns with international endeavours towards sustainable energy solutions and significantly contributes to the professional field's understanding of geothermal energy exploration and utilization. As we embark on the exploration of Egypt's geothermal potential in the Central Eastern Desert and Red Sea region, it is evident that the outcomes will extend beyond national boundaries. The findings of this research will impact the broader global discourse on renewable energy, reinforcing the importance of geothermal resources in achieving a sustainable and environmentally friendly energy future.

## Geological setting

**Figure 1:**
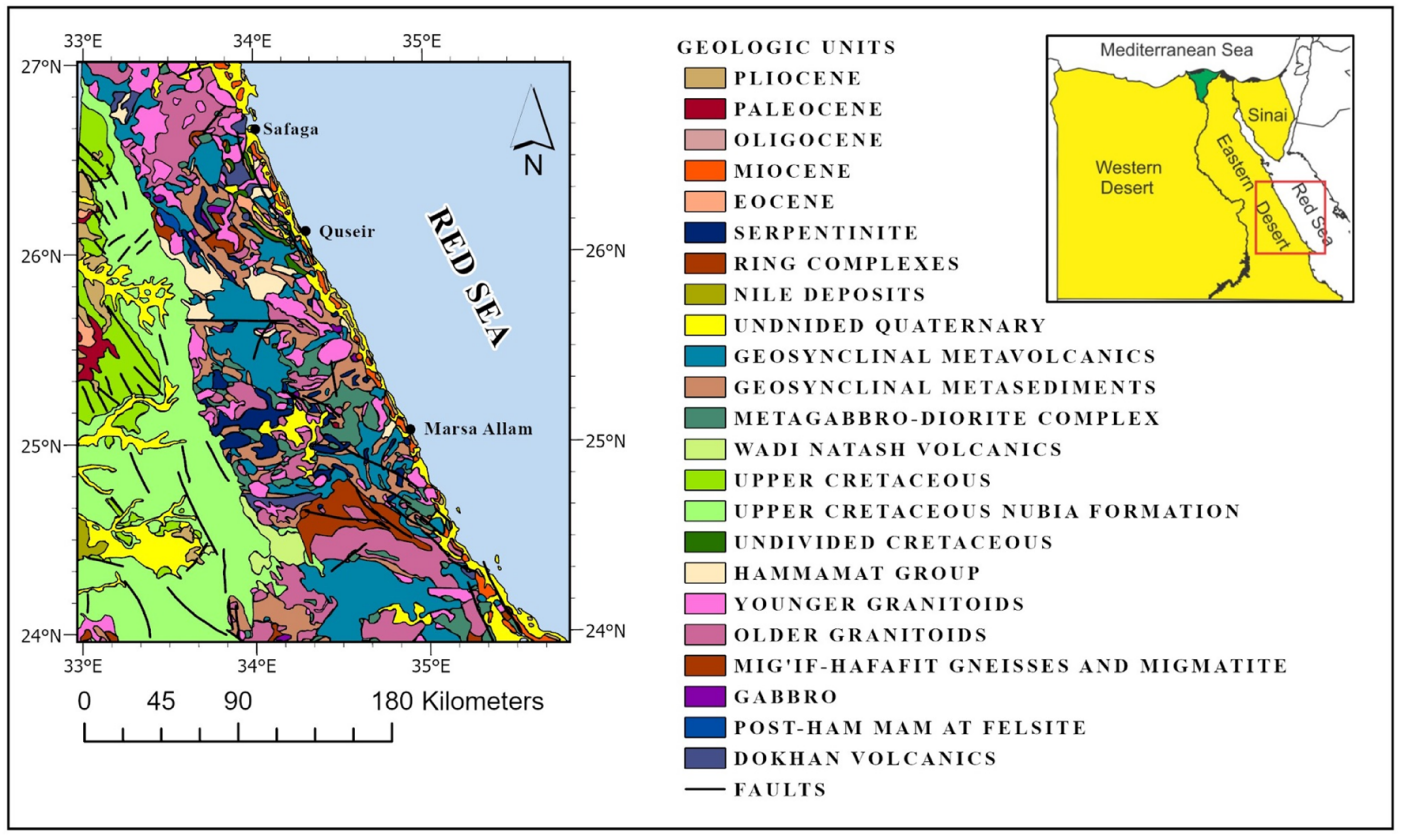
Geological map of Central Eastern Desert from the 1:2 million scale map ^13^.

Figure 1 illustrates the distribution of geological outcrops in our research area, situated within the Central Eastern Desert of Egypt. The region falls within the Nubian Shield, which spans from the Red Sea rift to the southern Sinai Peninsula. The Nubian Shield, formed between 800 and 600 million years ago, emerged in an island arc-accretion tectonic setting, splitting the shield into the Nubian and Arabian sectors^11,12^.

The Eastern Desert zone, along the western shores of the Red Sea and the southern part of the Sinai Peninsula, represents the Nubian Shield^13^. Rift tectonics within the active Red Sea led to the creation of horst and graben formations in the Eastern Desert, particularly near the western coast. Notably, two significant lineaments, the Trans African Lineament (TAL) and the Central African Lineament (CAL) intersect in the Red Sea region, indicating early tectonic activity in the area^14–16^.

The Eastern Desert is characterized by the coexistence of older (880–610 Ma) and younger (600–530 Ma) granites. The older granitic rocks, including diorite, monzonite, trondhjemite, and tonalite, were deposited during the Shaitan (850–800 Ma), Hafafit (760–710 Ma), and Meatiq (630 Ma) deformation stages^17–20^. The post-orogenic and synorogenic granites (600–530 Ma) are alkaline and peralkaline in composition and are found in the older crust along the strike-slip shear zones controlled by the TAL and CAL^17,21–24^.

Volcanic activity has played a significant role in the geological history of the Eastern Desert. Mesozoic volcanic activity occurred during the Jurassic-Early Cretaceous period (155–125 Ma), while the Late Cretaceous-Early Tertiary periods (90–60 Ma) marked another phase of volcanic activity^25^. Subsequent volcanic events took place during the Cenozoic and Paleocene epochs, followed by widespread tertiary basaltic volcanism in the Eocene, associated with the opening of the Red Sea^25^.

These volcanic activities, combined with the presence of various granitic formations and the influence of the Nubian Shield, contribute to the geological complexity and diversity of the Central Eastern Desert. Understanding the history and characteristics of these geological features is essential for comprehending the region's natural heritage and evaluating its resource potential.

## Data set

**Figure 2:**
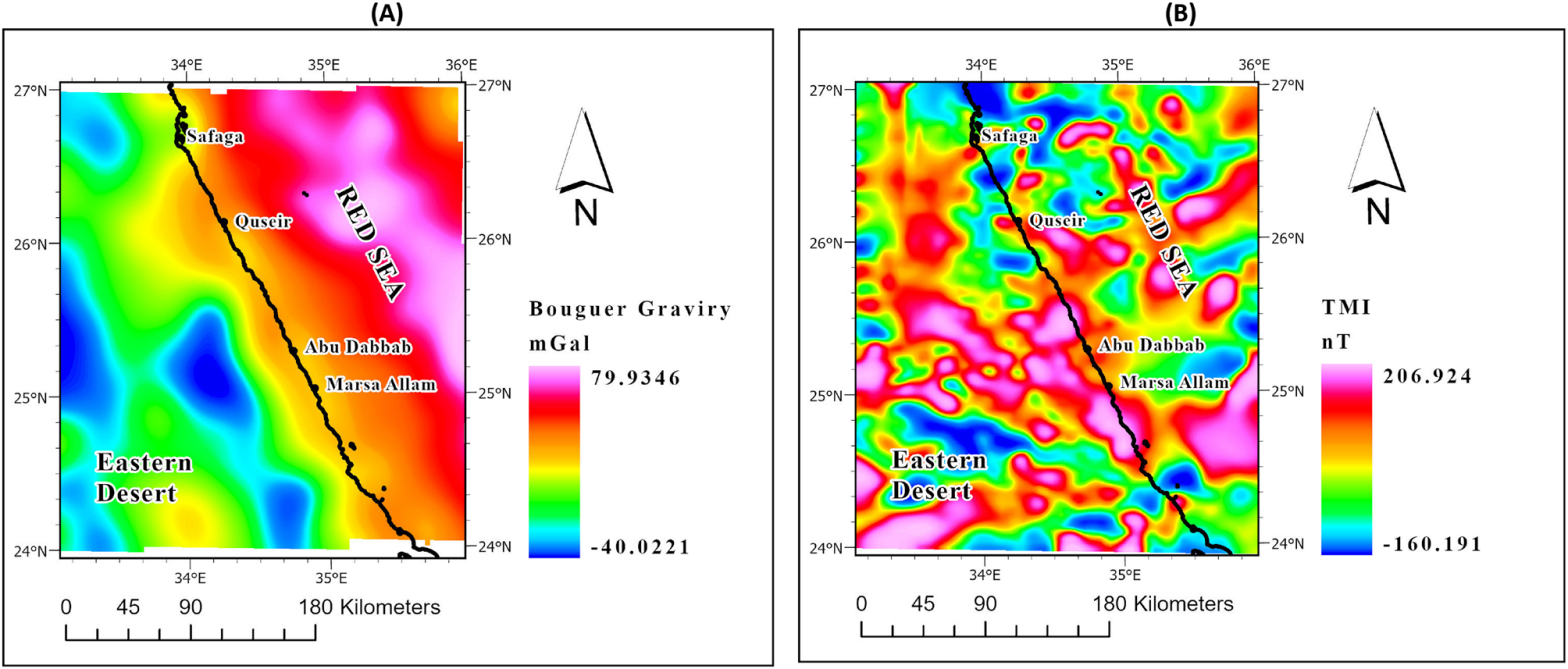
(**A**) Bouguer gravity map, (**B**) Total magnetic (TMI) map of the study area.

The Bouguer anomaly map, presented in Fig. 2A, was meticulously compiled by the esteemed Egyptian General Petroleum Corporation (EGPC) in 1985. This map, with its contour interval of 1 mGal and a scale of 1:100,000, provides a highly detailed representation of the data. Similarly, Fig. 2B showcases the magnetic map, prepared by the African Magnetic Mapping Project (AMMP), which surveyed with altitude elevation of 1 km along parallel flight lines.

In the pursuit of unlocking the geothermal potential of the entire study area, the magnetic data sourced from these maps have been harnessed. It is important to highlight that the magnetic dataset, a valuable resource in our research endeavours, has been obtained through the extensive African Magnetic Mapping Project (AMMP), sponsored by the esteemed organization Getech. The AMMP compilation has provided us with an invaluable, consistent 1-km grid of total magnetic intensity (TMI) data, a testament to the meticulousness and dedication of the project AMMP^26,27^.

By utilizing these meticulously compiled maps and datasets, we aim to contribute to the body of knowledge surrounding geothermal exploration in the study area. The richness and accuracy of these resources make them a cornerstone of our research, empowering us to delve into the geothermal potential with utmost precision and thoroughness.

## 3D Inversion modelling

Gravity and magnetic inversion are powerful geophysical techniques used to model subsurface structures based on gravity and magnetic field measurements. This methodology provides valuable insights into the distribution of density and magnetization within the Earth's crust. Pygimili, a Python library, offers comprehensive tools for conducting gravity and magnetic inversion studies.

The basic principle behind gravity inversion is that variations in subsurface density distribution can be inferred from gravity anomalies. The gravity anomaly is the difference between the observed gravity value and the theoretical gravity value, which is calculated based on the Earth's normal gravity field. By solving the inverse problem, which involves minimizing the difference between observed and predicted gravity anomalies, the density distribution can be estimated^28–30^.

In contrast, magnetic inversion primarily concerns the modelling of magnetization beneath the Earth's surface. Magnetic anomalies result from differences in the magnetic characteristics of rocks. The objective of magnetic inversion is to ascertain the arrangement of magnetization within the subsurface by reducing the mismatch between observed magnetic anomalies and their anticipated counterparts. This undertaking entails resolving a non-linear inverse problem.

Pygimili, a popular Python library for gravity and magnetic inversion, provides a range of functionalities for data processing, modelling, and inversion. It offers various inversion algorithms and regularization techniques to ensure stable and reliable results. Pygimili incorporates powerful numerical solvers and optimization algorithms to efficiently solve the inverse problems associated with gravity and magnetic inversion.

Equations (1 and 2) play a crucial role in gravity and magnetic inversion^28–30^. Some commonly used equations include Bouguer's gravity equation, which relates gravity anomalies to subsurface density variations, and the magnetic field equation, which relates magnetic anomalies to subsurface magnetization. These equations form the foundation for the forward modelling process in gravity and magnetic inversion.

In gravity inversion, one commonly used equation is Bouguer's gravity equation, which relates gravity anomalies to subsurface density variations:

1 $$ \Delta g = 2\pi G\rho \Delta z $$

where Δg is the observed gravity anomaly, G is the gravitational constant, ρ is the density contrast between the anomaly and the background, and Δz is the vertical distance between the anomaly and the observation point^28–30^.

In magnetic inversion, the equation used to relate magnetic anomalies to subsurface magnetization depends on the magnetic field measurement type. For total magnetic field anomalies, the equation is:

2 $$ \Delta B = {\raise0.7ex\hbox{${\mu 0}$} \!\mathord{\left/ {\vphantom {{\mu 0} {4\pi }}}\right.\kern-0pt} \!\lower0.7ex\hbox{${4\pi }$}} * \int {\left( {M \cdot \nabla \left( {{\raise0.7ex\hbox{$1$} \!\mathord{\left/ {\vphantom {1 r}}\right.\kern-0pt} \!\lower0.7ex\hbox{$r$}}} \right)} \right)} dV $$

where ΔB is the observed magnetic anomaly, μ0 is the magnetic permeability of free space, M is the magnetization vector, ∇ is the gradient operator, r is the distance from the magnetization source, and the integral is taken over the volume (V) of the subsurface^28–30^.

It's important to note that these equations represent the forward modelling process, where the observed anomalies are calculated based on the assumed density or magnetization models. In the inversion process, these equations are used inversely to estimate the density or magnetization distribution by minimizing the difference between observed and predicted anomalies.

The inversion process in gravity and magnetic inversion incorporates several key constraints to ensure accurate results. These constraints are carefully designed to guide the inversion process and produce reliable models. The main constraints employed are depth weights, absolute error, and lambda values.

Depth weights play a vital role in the inversion process by imposing a constraint based on the boundary depths of the grid. These weights are calculated within the code, with deeper boundaries assigned lower weights, indicating a weaker constraint. By normalizing the depth weights, they are brought within a consistent range, ensuring their effectiveness in the inversion process.

Another crucial constraint is the absolute error, which is determined using the standard deviation of the observed data. This error represents the acceptable deviation between the inverted model response and the actual data. To control the tolerance level of the inversion, a multiplier (typically set at 0.1) can be adjusted. By fine-tuning this multiplier, the inversion process can be tailored to meet specific accuracy requirements.

Lambda values form an integral part of the inversion process, as the code performs a series of inversions with different lambda (λ) values. Lambda serves as a regularization parameter, striking a balance between fitting the observed data and maintaining simplicity or smoothness in the inverted model. By varying the lambda values, the inversion process applies different levels of regularization, resulting in models with varying degrees of complexity. This flexibility allows for the exploration of different trade-offs between data fit and model simplicity, enabling the creation of models that best capture the underlying geological features.

Overall, the inclusion of these constraints in the gravity and magnetic inversion process enhances its reliability and flexibility, enabling accurate modelling of subsurface structures while considering the inherent uncertainties and complexities associated with the inversion process.

Please keep in mind that various inversion methods and variations depend on the specific problem and assumptions made. The equations presented here provide a basic understanding of the relationship between observed anomalies and subsurface properties in gravity and magnetic inversion.

### Bouguer gravity inversion

**Figure 3:**
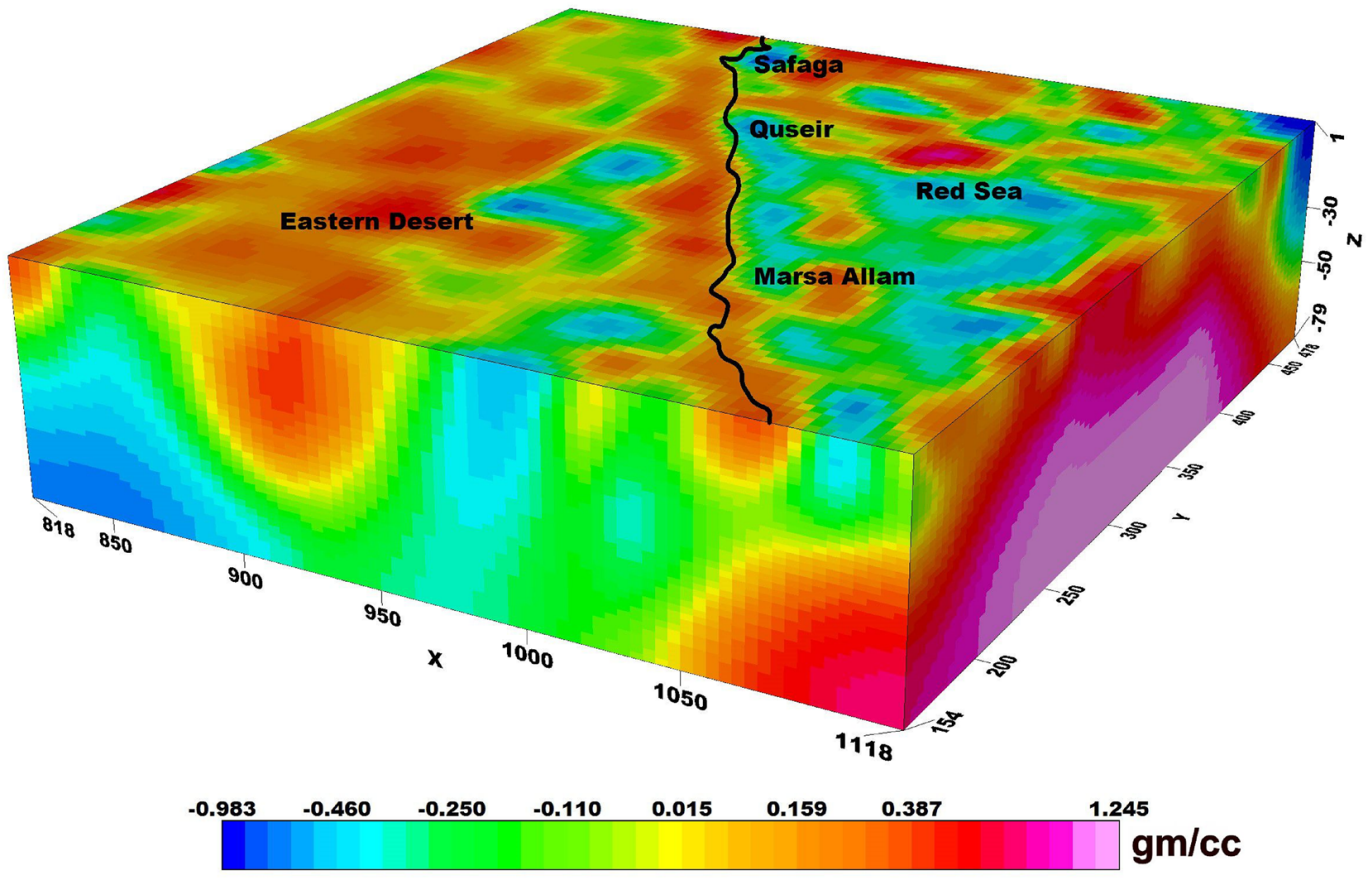
3D density inversion model of the study area.

The application of 3D gravity inversion modelling using the Pygimli library in Python to investigate the subsurface structures of the Central Eastern Desert and Red Sea region is shown in Fig. 3. Bouguer gravity data, with a cell size of 4 km in both the X and Y dimensions, and 2 km in the Z dimension, are utilised for the inversion process. The modelling depth is extended up to 80 km to capture a comprehensive understanding of the geological features and potential mineral deposits in the study area. By implementing the Pygimli library, which offers a powerful and customisable framework for gravity inversion, this research aims to provide valuable insights into the subsurface density variations and geological structures of the Central Eastern Desert and Red Sea region.

The density inversion process employs specific parameters to calculate depth weight, with z_0_ set at 40 kms. The absolute error associated with this inversion is measured at 4.134%. Additionally, the range of lambda values utilized in the process spans from 100 to 1000. Lastly, the initial model employed for the inversion is set at 0.1.

The 3D density inversion modelling in our study area revealed a significant range of density contrast values, ranging from -0.93 to 1.21 gm/cc, with a background density of 2.67 gm/cc. These density variations offer crucial information regarding the subsurface composition and potential geological structures within the Central Eastern Desert and Red Sea region. The inversion results provide a detailed understanding of the density distribution along our study area, shedding light on potential mineral deposits and geological anomalies that may contribute to the region's economic significance. The integration of the Pygimli library, with its robust and customizable capabilities, ensures the accuracy and reliability of the inversion results, thereby enhancing our understanding of the subsurface dynamics in this complex geological setting.

**Figure 4:**
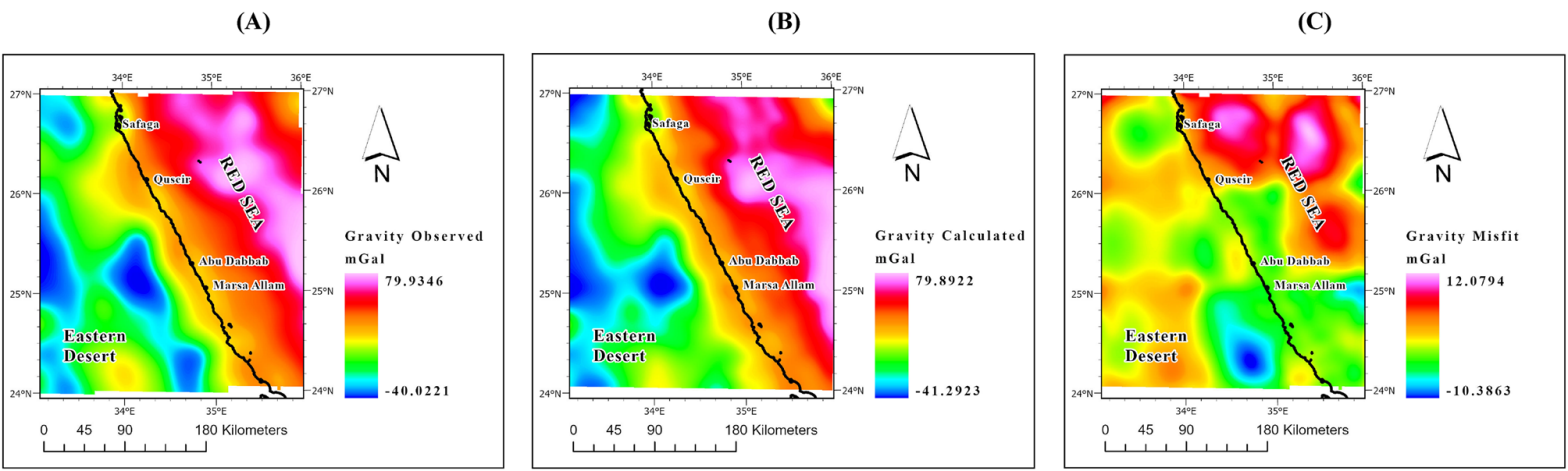
(**A**) Observed, (**B**) Calculated, and (**C**) Misfit maps of the study area resulting from the 3D gravity inversion model.

The gravity anomaly maps (Fig. 4), comprising observed, calculated, and misfit data, serve as a robust validation tool for assessing the quality of the inversion model. These maps provide comprehensive coverage of the entire study area, offering precise and intricate details. By comparing the observed values with the calculated ones and analyzing the resulting misfit, this evaluation method ensures the accuracy and reliability of the inversion model.

### Magnetic inversion

**Figure 5:**
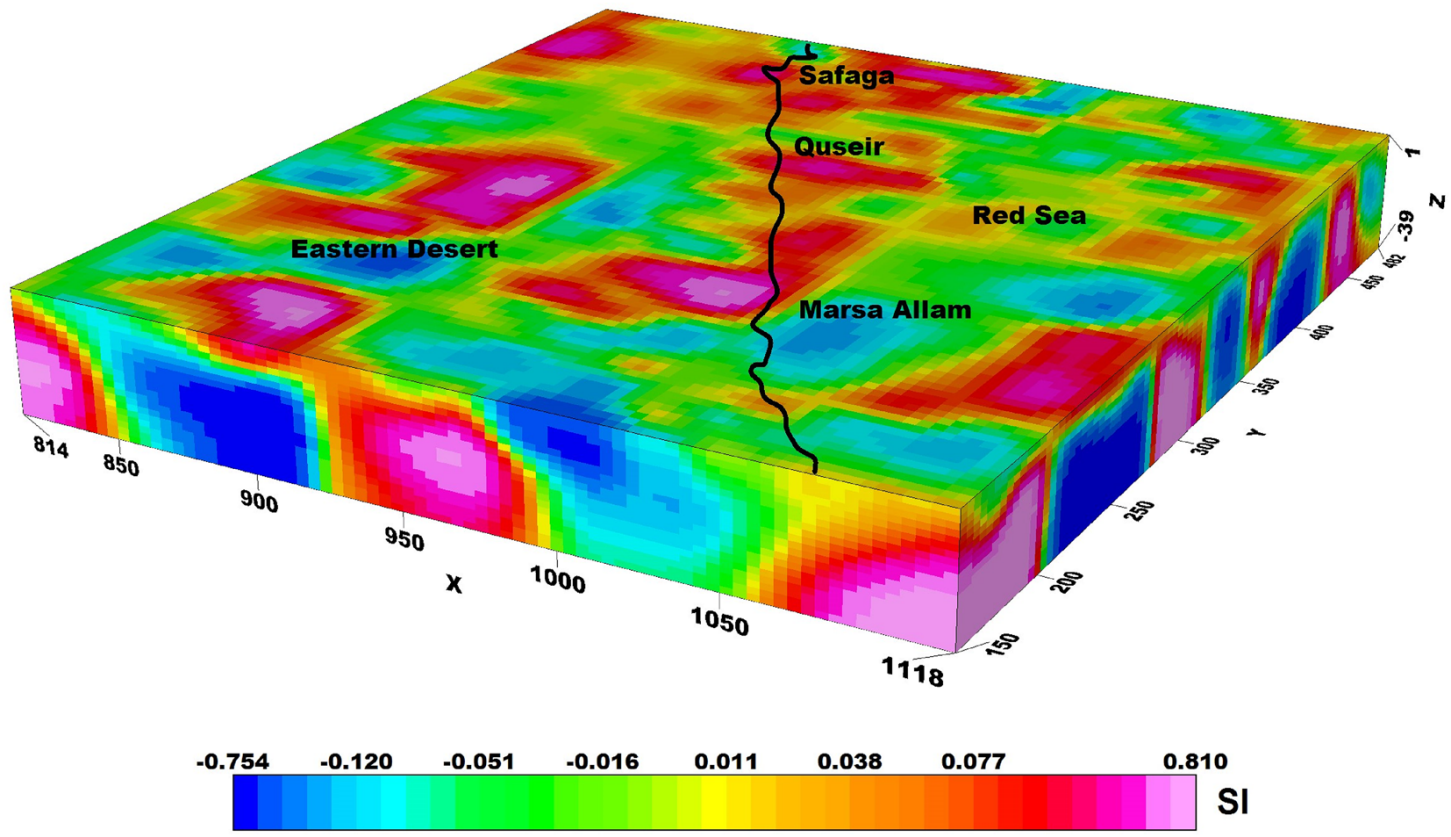
magnetic data inversion model of the study area.

The Pygimli library in Python was also utilised to conduct 3D magnetic inversion modelling, enabling an investigation into the subsurface structures of the Central Eastern Desert and Red Sea region (Fig. 5). Similar to gravity inversion, the magnetic inversion process utilised data with a cell size of 4 km in both the X and Y dimensions and 2 km in the Z dimension. The magnetic data used for the inversion was collected through magnetic surveys and measured the magnetic field intensity.

In this study, the modelling depth for magnetic inversion was also extended up to 40 km to capture a comprehensive understanding of the geological features and potential mineral deposits in the study area. By employing the Pygimli library, which provides a powerful and customisable framework for magnetic inversion, this research aims to offer valuable insights into the subsurface magnetic properties and geological structures of the Central Eastern Desert and Red Sea region.

The magnetic inversion process employs specific parameters to calculate depth weight, with z_0_ set at 20 kms. The absolute error associated with this inversion is measured at 8.46%. Additionally, the range of lambda values utilized in the process spans from 1000 to 10000. Lastly, the initial model employed for the inversion is set at 0.001.

The 3D magnetic inversion modelling in our study area unveiled a broad range of magnetic susceptibility values, spanning from -0.53 to 0.49 SI. These susceptibility variations provide crucial information regarding the subsurface magnetic properties and potential geological structures within the study area. The inversion results offer a detailed understanding of the magnetic distribution along our study area, shedding light on potential mineral deposits and geological anomalies that could hold economic significance for the region.

The integration of the Pygimli library, with its robust and customizable capabilities, ensures the accuracy and reliability of the magnetic inversion results. This integration enhances our understanding of the subsurface dynamics in this complex geological setting, facilitating further exploration and characterization of the Central Eastern Desert and Red Sea region's magnetic properties and geological structures.

**Figure 6:**
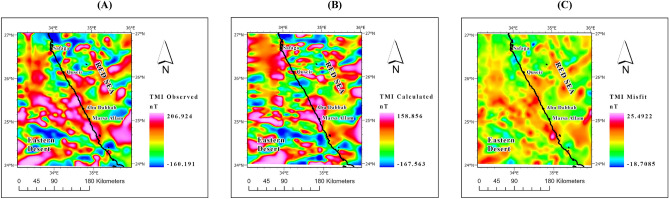
(**A**) Observed, (**B**) Calculated, and (**C**) Misfit maps of the study area resulting from the 3D magnetic inversion model.

The magnetic anomaly maps (Fig. 6), comprising observed, calculated, and misfit data, serve as a robust validation tool for assessing the quality of the inversion model. These maps provide comprehensive coverage of the entire study area, offering precise and intricate details. By comparing the observed values with the calculated ones and analyzing the resulting misfit, this evaluation method ensures the accuracy and reliability of the inversion model.

### Calculating 3D temperature model

Temperature plays a crucial role in understanding the thermal structure and dynamics of the Earth's interior. Accurate temperature models are essential for various scientific disciplines, including geophysics, geodynamics, and mineral exploration. In this essay, we discuss the construction of a temperature model for a specific study area by utilizing the WINTERC-G model and coupling it with inversion techniques.

The WINTERC-G model, as described by Fullea et al.^31^, provides a comprehensive framework for mapping the upper mantle thermochemical heterogeneity. This model integrates seismic waveforms, heat flow, surface elevation, and gravity satellite data to derive density variations, which are then correlated with temperature.

To construct a temperature model for our study area, we adopted the density-temperature relation from the WINTERC-G model. We employed inversion techniques to extract the density model based on available geophysical data and replaced the model density values with our inversion density values. By utilizing this modified density model, we calculated the corresponding temperature distribution for the study area.

**Figure 7:**
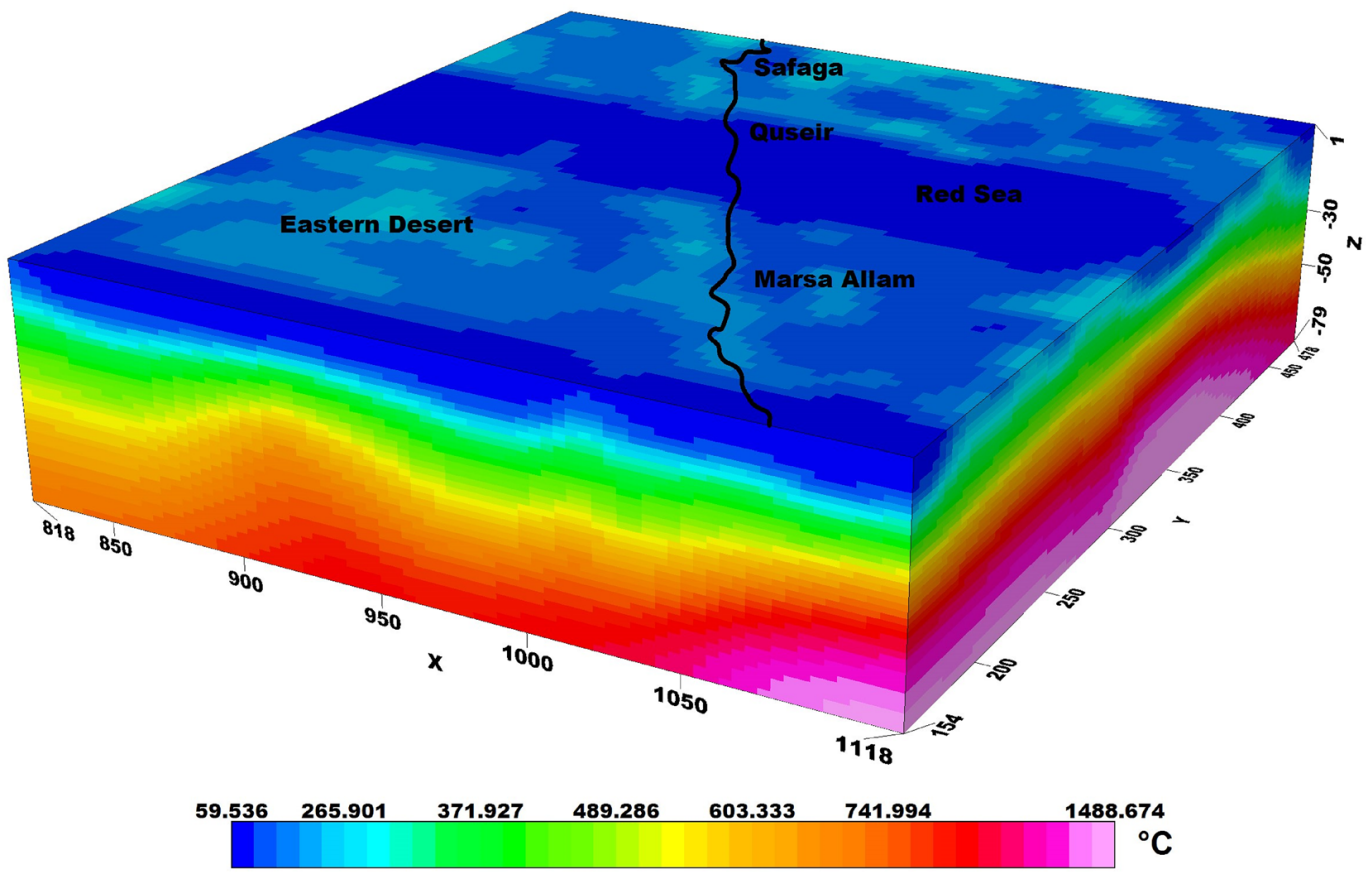
Temperature model of the study area.

The temperature model obtained through this approach provides valuable insights into the thermal structure of the study area (Fig. 7). It reveals variations in temperature at different depths reaching a depth of 80 km and the temperature values ranged from 59 to 1488 °C, allowing for a better understanding of subsurface processes and the potential presence of thermal anomalies.

The integration of the WINTERC-G model and inversion techniques and temperature wells published by Morgan et al.^32^ enables us to refine the temperature model by incorporating local density variations derived from inversion methods. This enhances the accuracy and reliability of the temperature estimations, providing a more realistic representation of the subsurface thermal conditions.

## Result and discussion

The Bouguer gravity map utilized in this study (Fig. 2A) offers a comprehensive depiction of gravity anomalies, covering a wide range of values from -47 to 104 mGal. Of particular interest is the distinct contrast in gravity values between the Red Sea and the onshore region of the Eastern Desert.

The Red Sea exhibits higher gravity values compared to the onshore area of the Eastern Desert. This discrepancy can be attributed to two primary factors. Firstly, the higher gravity values in the Red Sea can be explained by the presence of the oceanic crust. It is well-established that oceanic crust tends to be denser than continental crust, resulting in elevated gravity values in regions characterized by oceanic crust.

Conversely, the lower gravity values observed in the Eastern Desert can be attributed to two factors. Firstly, the continental crust is generally less dense compared to the oceanic crust, leading to relatively lower gravity values in continental regions. Additionally, the occurrence of fractures in the Eastern Desert plays a significant role. The presence of fractures, which are frequent in this region, contributes to the reduction in gravity values as fractures tend to contain less dense materials.

These gravity variations observed in the study area provide valuable insights into the geological characteristics of the Red Sea and the Eastern Desert. The higher gravity values in the Red Sea can be linked to the presence of the oceanic crust, suggesting potential tectonic activity and associated geological processes. On the other hand, the lower gravity values in the Eastern Desert can be attributed to both the less dense continental crust and the prevalence of fractures.

The 3D density inversion model employed in this study (Fig. 3) provides valuable insights into the subsurface density distribution, revealing density contrast values ranging from -0.9 to 1.2 gm/cc. Notably, the model highlights distinct variations in density within the study area, particularly in the northern part of the Red Sea.

A closer examination of the model reveals the presence of highly dense zones near the surface of the region. As one moves deeper towards the center of the Red Sea, the density progressively increases. This observation suggests a significant variation in density with depth, indicative of underlying geological structures and processes.

In contrast, along the onshore line, the density exhibits an intriguing pattern. The surface density is comparatively higher, gradually decreasing with increasing depth. This phenomenon can be attributed to a higher concentration of crustal fractures in this area, resulting in a lower overall density beneath the surface.

The Marsa Allam and Abu-Dabbab areas, as well as the region between Safaga and Qusier, exhibit a distinct high-density characteristic, which can be attributed to their association with the deep Red Sea. The density inversion model analysis supports the hypothesis that these areas experience elevated temperatures due to heat transfer from the Red Sea.

**Figure 8:**
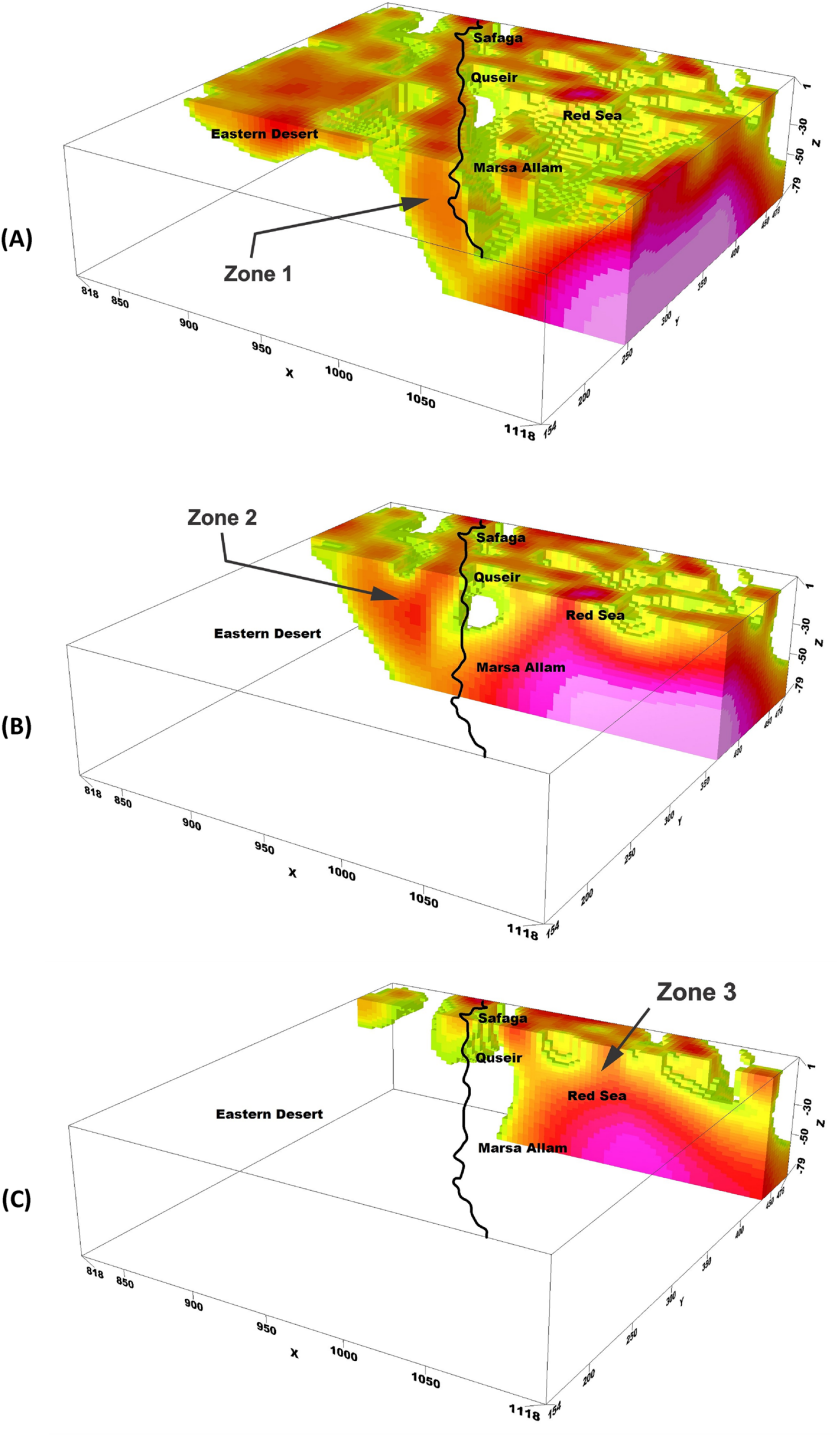
The three interesting zones of geothermal energy prospecting shown from the density inversion model in the study area.

The observed high-density zones align with the density deep Red Sea, as depicted in the inversion model with arrangement (Fig. 8A,B). This spatial correlation suggests a potential connection between the density distribution and the transfer of heat from the Red Sea to these specific areas.

The inversion model analysis provides an understanding of the thermal dynamics within the study region. The transfer of heat is likely responsible for the elevated temperatures observed in Marsa Allam, Abu-Dabbab, and the region between Safaga and Qusier.

In another noteworthy area within the study region, located in the northern part of the Red Sea (Fig. 8C), distinct high-density zones are observed. These zones can be attributed to the presence of areas with elevated heat flow. This connection is supported by previous heat flow measurements conducted by Martínez & Cochran and Sherif et al. ^33,34^.

The high-density zones observed in the northern Red Sea region are closely linked to areas characterized by high heat flow. These heat flow measurements, as reported by Martínez & Cochran and Sherif et al.^33,34^, provide valuable evidence for the association between increased heat transfer and the resulting density variations in this area.

Furthermore, the magnetic data inversion technique (Fig. 5) was employed to portray the distribution of magnetic susceptibility beneath the surface. The calculated magnetic susceptibility values range from -0.5 to 0.4 SI. Notably, a substantial portion of the inverted data reveals values equal to zero or less, indicating the absence of magnetic susceptibility in these areas.

The presence of magnetic susceptibility values equal to zero or less holds significance, as it suggests a lack of magnetic response in these regions. This absence of magnetic susceptibility can be attributed to high temperatures, which result in the loss of magnetization. Elevated temperatures can demagnetize materials, thereby rendering them non-responsive to magnetic fields.

The occurrence of areas with no magnetic susceptibility, indicated by values equal to zero or less, provides valuable insights into the thermal conditions within the study area. It suggests the presence of regions where high temperatures have essentially demagnetized the materials, leading to the loss of their magnetic properties.

**Figure 9:**
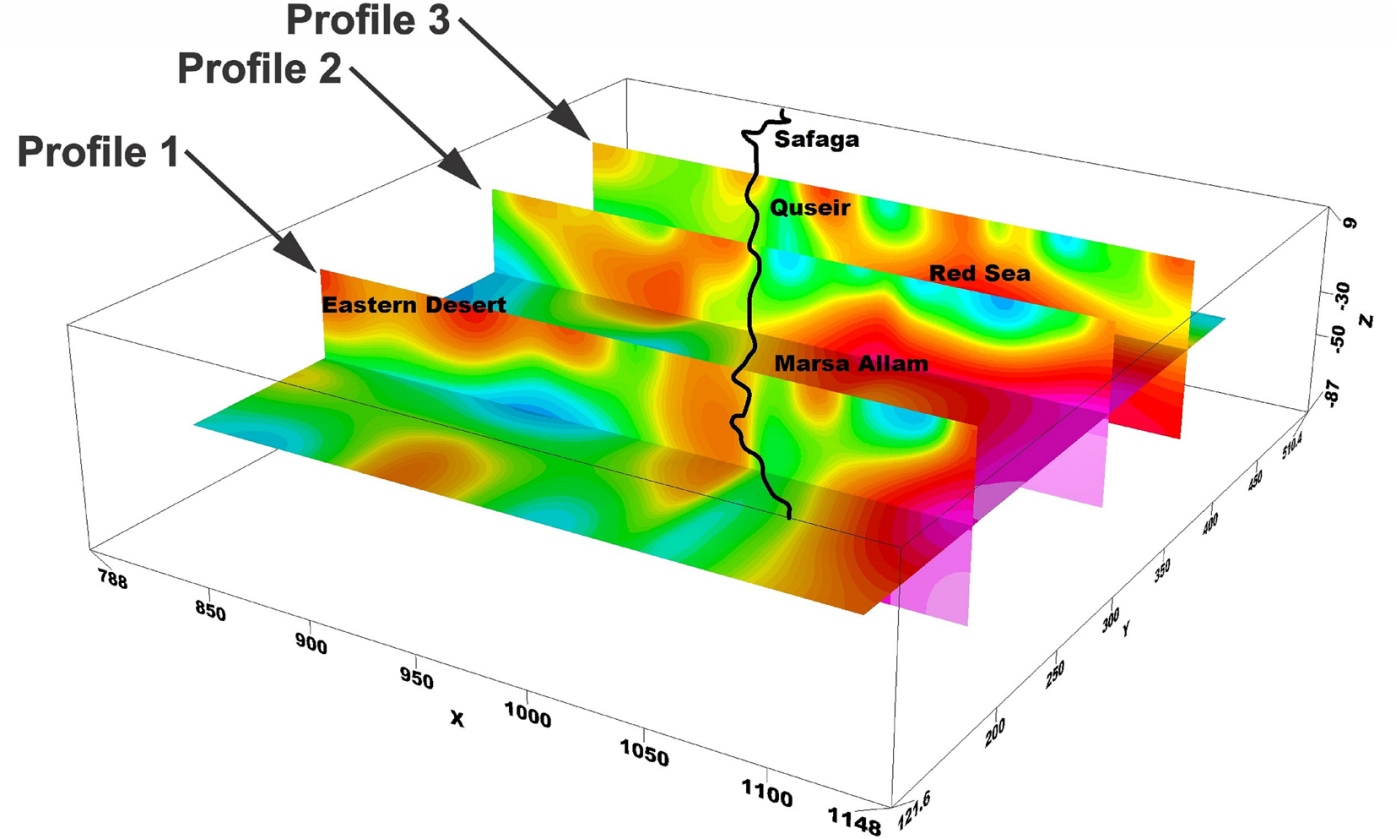
Profiles location of the promising geothermal potentialities of the study area.

**Figure 10:**
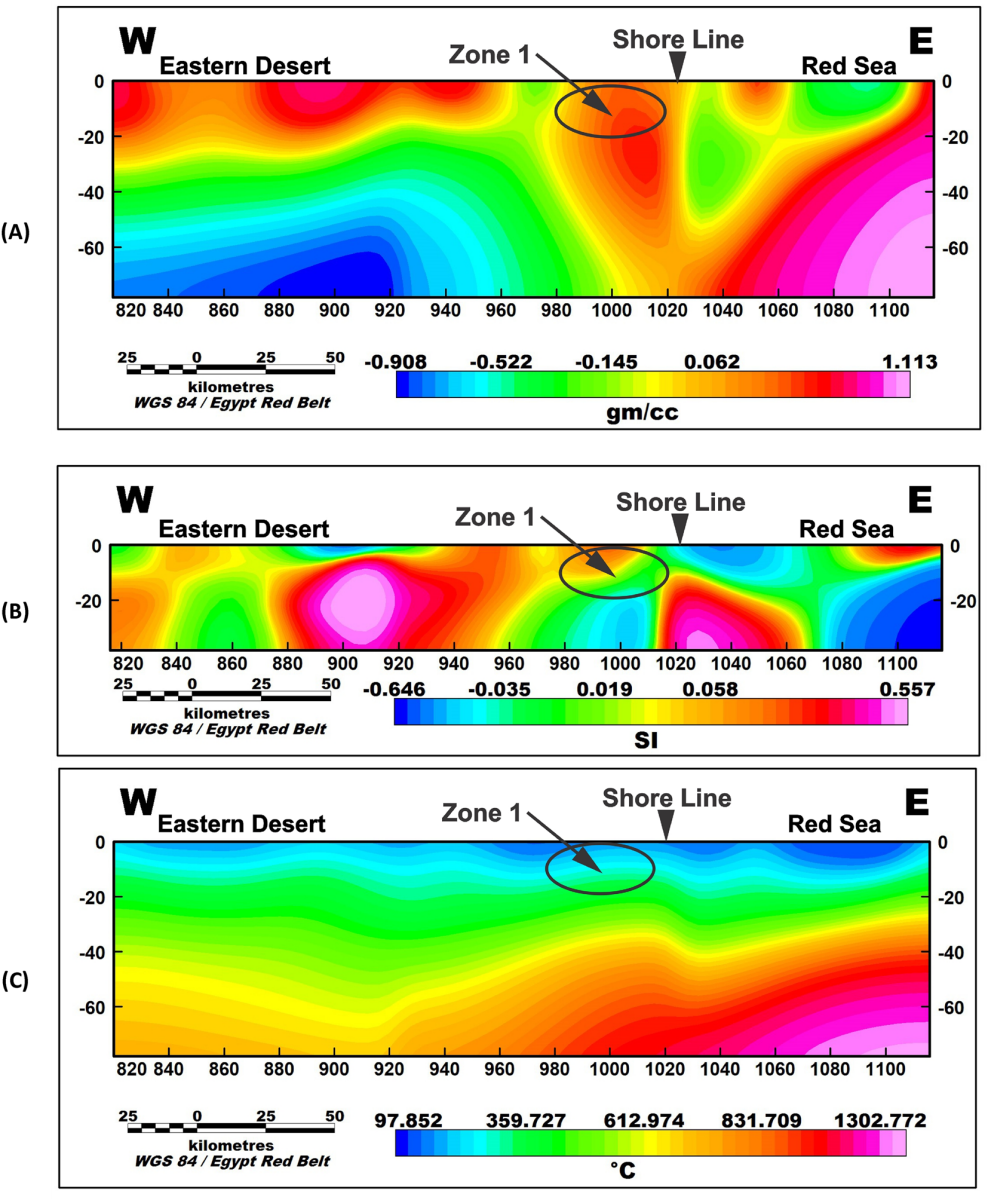
Profile (1) of the area between Marsa Allam, Abu-Dabbab where (**A**) gravity inversion profile, (**B**) magnetic inversion profile and (**c**) temperature profile.

**Figure 11:**
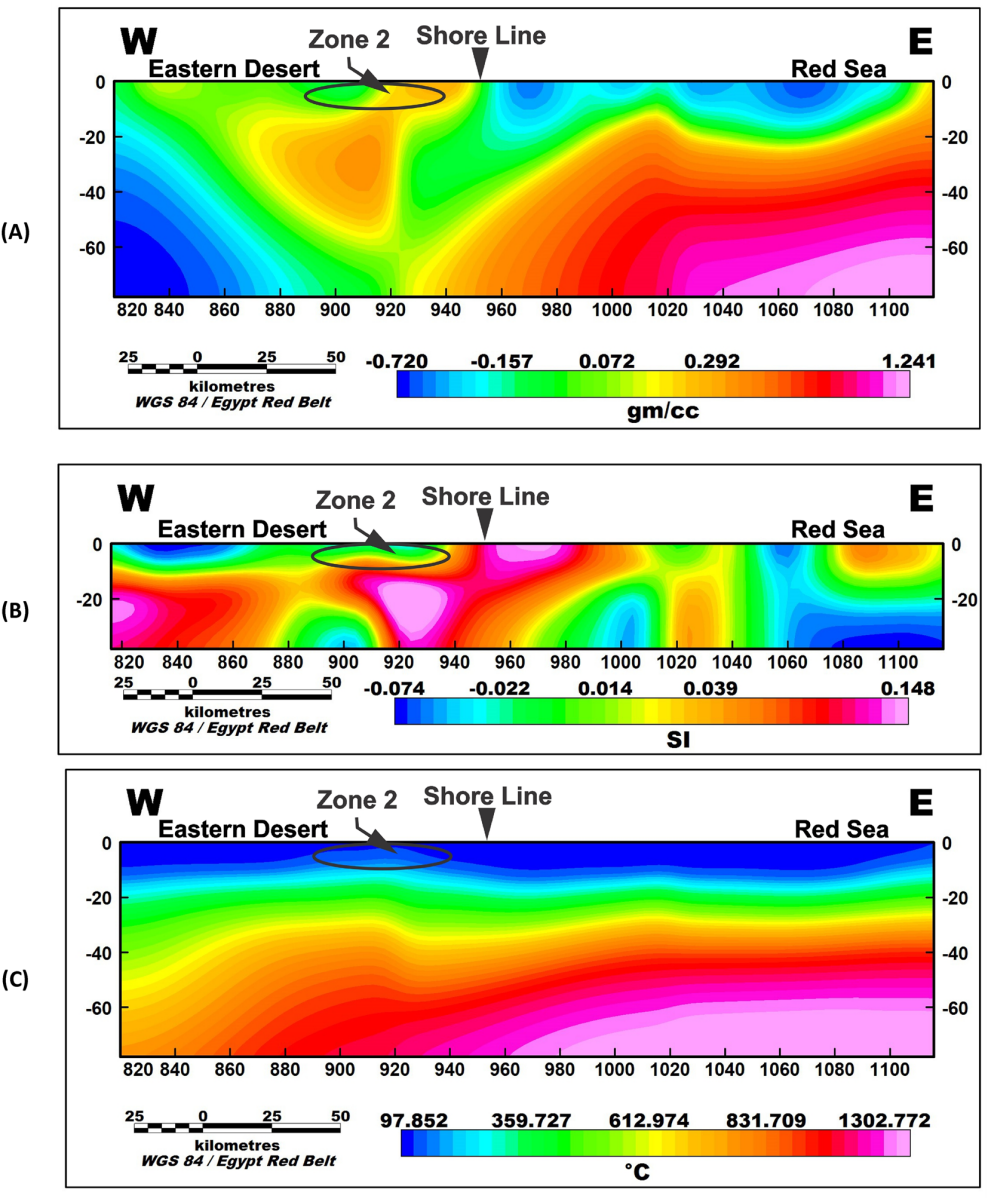
Profile (2) of the area between Safaga and Qusier where (**A**) gravity inversion profile, (**B**) magnetic inversion profile and (**c**) temperature profile.

**Figure 12:**
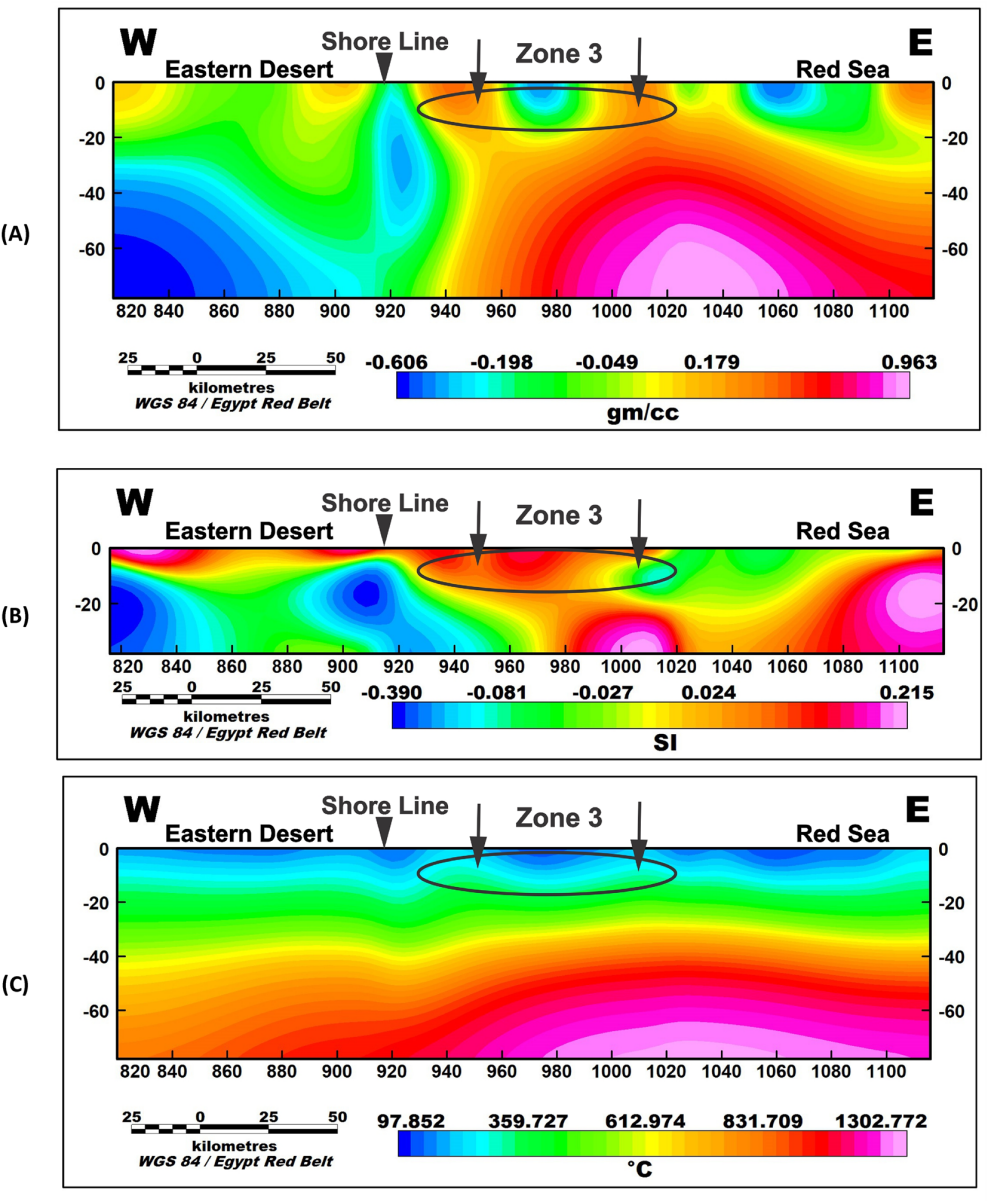
Profile (3) of the North Red Sea in the study area where (**A**) gravity inversion profile, (**B**) magnetic inversion profile and (**c**) temperature profile.

To further investigate the potential geothermal resources within the study area, three profiles were taken along the promising area (Fig. 9). These profiles encompass gravity, magnetic inversion, and temperature models as shown in Figs. 10, 11, and 12. The first zone includes the Marsa Allam and Abu-Dabbab areas (Fig. 10), followed by the second zone located between Safaga and Qusier (Fig. 11). The third zone lies to the north of the Red Sea (Fig. 12). Through a comparative analysis of the density and magnetic susceptibility profiles spanning these zones in an east–west direction, a notable correlation between high-density areas and low magnetic susceptibility values was observed. This correlation supports the proposition that these high-density regions are indicative of elevated temperatures, likely associated with heat transfer from the Red Sea.

The compatibility observed between the high-density areas and low magnetic susceptibility values substantiates the hypothesis regarding the presence of higher temperatures. The concurrence of high density and low magnetic susceptibility implies that these regions experience enhanced thermal conditions, which can be attributed to the transfer of heat from the Red Sea.

The profiles from the temperature model, as shown in Figs. 10C, 11C, and 12C, reveal an increasing temperature in these zones nearing the surface. The findings from this comparative analysis provide compelling evidence in support of the suggestion that the correlation between high-density areas and low magnetic susceptibility values strengthens the notion of temperature transfer from the Red Sea as a contributing factor to the observed geothermal potential in these zones.

**Figure 13:**
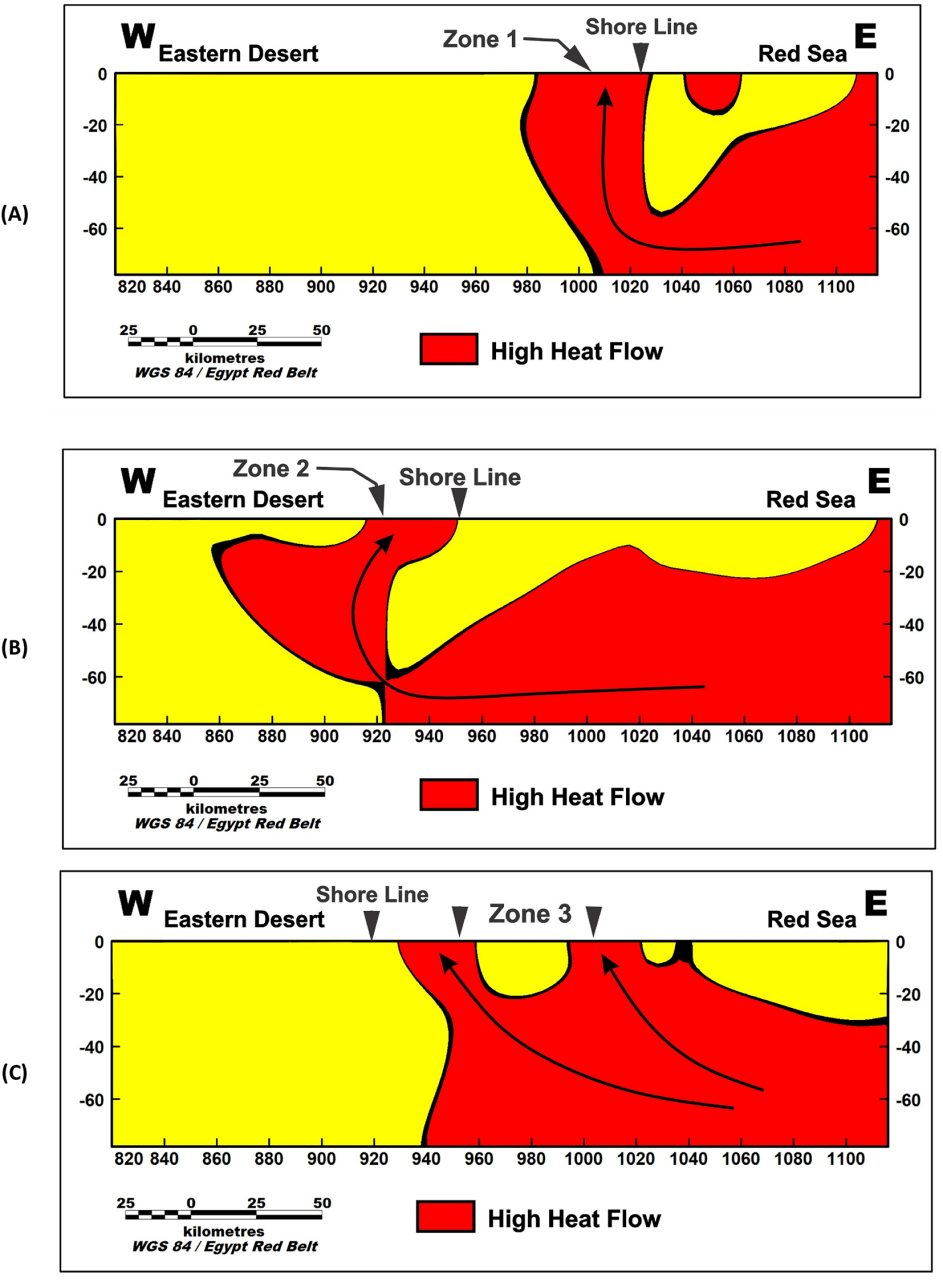
Illustrates a simulated depiction of the migration pathway of heat flow originating from the Red Sea towards the identified areas of geothermal potential.

Figure 13 offers a compelling visual representation, showcasing a simulated depiction of the migration pathway through which thermal energy emanates from the Red Sea and traverses towards the meticulously identified zones of interest with substantial geothermal potential. This visualization vividly portrays the intricate process of heat transfer, shedding light on the remarkable journey of thermal energy from its source to the targeted areas where its utilization for geothermal purposes holds immense promise.

## Conclusion

Based on the comprehensive analysis of the Bouguer gravity map, 3D gravity inversion model, and magnetic data inversion, several conclusions can be drawn:

1. The 3D density inversion model reveals distinct variations in density within the study area. Highly dense zones are observed near the surface of the Red Sea, indicating a significant variation in density with depth and underlying geological structures and processes.
2. Along the onshore line, the density gradually decreases with increasing depth, likely due to a higher concentration of crustal fractures in the area.
3. The Marsa Allam and Abu-Dabbab areas, as well as the region between Safaga and Qusier, exhibit high-density characteristics associated with the deep Red Sea. This suggests a connection between the density distribution and the transfer of heat from the Red Sea to these areas, leading to elevated temperatures.
4. In the northern part of the Red Sea, high-density zones are observed, which can be attributed to areas with elevated heat flow. This supports the association between increased heat transfer and density variations in this region.
5. The magnetic data inversion technique reveals the absence of magnetic susceptibility in certain areas, indicating high temperatures that have demagnetized the materials.
6. The correlation between high-density areas and low magnetic susceptibility values further supports the proposition of elevated temperatures and heat transfer from the Red Sea.
7. Comparative analysis of the temperature profiles confirms increasing temperatures nearing the surface in the promising zones, providing compelling evidence for the geothermal potential associated with heat transfer from the Red Sea.

Overall, the study suggests that the Red Sea region exhibits geological characteristics and thermal dynamics that contribute to its geothermal potential. The correlation between gravity, magnetic susceptibility, and temperature profiles strengthens the understanding of heat transfer processes and identifies areas with significant geothermal resources.

## Data Availability

The data that support the findings of this study are available from the corresponding author upon reasonable request.
